# Homeostatic response under carcinogen withdrawal, heme oxygenase 1 expression and cell cycle association

**DOI:** 10.1186/1471-2407-6-286

**Published:** 2006-12-14

**Authors:** Cynthia C Castronuovo, Paula A Sacca, Roberto Meiss, Fabiana A Caballero, Alcira Batlle, Elba S Vazquez

**Affiliations:** 1 Departamento de Química Biológica, Facultad de Ciencias Exactas y Naturales, Universidad de Buenos Aires, Ciudad Universitaria, Pabellón II - 4to Piso, (1428) CONICET Buenos Aires, Argentina; 2 Centro de Investigaciones sobre Porfirinas y Porfirias (CIPYP), CONICET, Hospital de Clínicas, Universidad de Buenos Aires, Buenos Aires, Argentina; 3 Departamento de Patología, Instituto de Estudios Oncológicos, Academia Nacional de Medicina, Pacheco de Melo 3081 - (1425) Buenos Aires, Argentina

## Abstract

**Background:**

Chronic injury deregulates cellular homeostasis and induces a number of alterations leading to disruption of cellular processes such as cell cycle checkpoints and apoptosis, driving to carcinogenesis. The stress protein heme oxygenase-1 (HO-1) catalyzes heme degradation producing biliverdin, iron and CO. Induction of HO-1 has been suggested to be essential for a controlled cell growth. The aim of this work was to analyze the *in vivo *homeostatic response (HR) triggered by the withdrawal of a potent carcinogen, p-dimethylaminoazobenzene (DAB), after preneoplastic lesions were observed. We analyzed HO-1 cellular localization and the expression of HO-1, Bcl-2 and cell cycle related proteins under these conditions comparing them to hepatocellular carcinoma (HC).

**Methods:**

The intoxication protocol was designed based on previous studies demonstrating that preneoplastic lesions were evident after 89 days of chemical carcinogen administration. Male CF1 mice (n = 18) were used. HR group received DAB (0.5 % w/w) in the diet for 78 days followed by 11 days of carcinogen deprivation. The HC group received the carcinogen and control animals the standard diet during 89 days. The expression of cell cycle related proteins, of Bcl-2 and of HO-1 were analyzed by western blot. The cellular localization and expression of HO-1 were detected by immnunohistochemistry.

**Results:**

Increased expression of cyclin E/CDK2 was observed in HR, thus implicating cyclin E/CDK2 in the liver regenerative process. p21^cip1/waf1 ^and Bcl-2 induction in HC was restituted to basal levels in HR. A similar response profile was found for HO-1 expression levels, showing a lower oxidative status in the carcinogen-deprived liver. The immunohistochemical studies revealed the presence of macrophages surrounding foci of necrosis and nodular lesions in HR indicative of an inflammatory response. Furthermore, regenerative cells displayed changes in type, size and intensity of HO-1 immunostaining.

**Conclusion:**

These results demonstrate that the regenerative capacity of the liver is still observed in the pre-neoplastic tissue after carcinogen withdrawal suggesting that reversible mechanism/s to compensate necrosis and to restitute homeostasis are involved.

## Background

Primary liver carcinoma or hepatocellular carcinoma (HC) constitutes the most common visceral malignant tumor and in some populations is the most common overall [1]. It arises, mainly, from hepatocytes, the major cell type of liver and it can be also derived from characteristic morphological cells named "oval cells" after the exposure to hepatocarcinogens, by an irreversible blockage in the process of normal differentiation and by generation of immortal transformed cells with proliferative potential. Dedifferentiation of normal mature hepatocytes is also accepted as a theory to postulate that a random clonal, near 20%, origin of HC is seen in experimental rat chemical hepatocarcinogenesis [2].

There are several models available to study the mechanisms of development of liver cancer *in vivo*. Cancer development involves both genotoxic and cell proliferative stages. Cell proliferation is essential for the carcinogenic process, not only to induce heritable damage to the DNA sequence during the initiation of cancer, but also to clonal expand these initiated cells during promotion [3]. There are several different methods to induce hepatic cell proliferation, among them partial hepatectomy, the use of a necrogenic dose of an initiator and fasting/refeeding with a subnecrogenic dose of initiator [3].

The liver has two main capacities, one is to regenerate after injury or resection [4] and the other is to self-regulate its growth and final mass development [5]. However, a reduction in liver cell mass, due to cell loss and/or cell atrophy, triggers a rapid regenerative response tailored to replace the lost tissue, resulting in an almost complete restitution of hepatic mass and function [6]. Partial hepatectomy triggers hepatocyte proliferation whereas excessive liver mass is regulated by apoptosis [7]. In addition to hepatocytes and non-parenchymal cells, the liver contains "stem" cells which can generate a transitory compartment of precursor hepatocytes, named oval cells [5,8].

Cell cycle regulators controlling G_1 _phase progression are frequently involved in the carcinogenesis of many human cancer types [9]; even of HC [10]. There are at least 9 cdks. These kinases are activated by D-type cyclins (D1, D2, D3) and cyclin E, and inhibited by two families of cdk inhibitors, the INK and Cip/Kip families [11].

Aberrant cell proliferation and abnormal expression of cell cycle related proteins are observed in inflammatory disorders. Cellular over expression of heme oxygenase-1 (HO-1), the anti-inflammatory and anti-oxidative stress protein that catalyzes heme degradation producing biliverdin, iron and CO, up-regulates p21^cip1/waf1^, diminishes cell growth proliferation and confers marked resistance to apoptosis. Consequently up-regulation of p21^cip1/waf1 ^contributes to the altered pattern of cell growth and resistance to apoptosis [12].

The multistep process of liver regeneration comprises at least two critical phases: the transition of the quiescent hepatocyte into the cell cycle (priming) and the progression beyond the restriction point in the G_1 _phase. The priming phase is characterized by the expression of immediate early genes which causes secondary activation of multiple genes and plays an important role in the initiation of hepatic regeneration. Activation of proto-oncogenes in the immediate early gene response involves both transcriptional and post-transcriptional mechanisms [7].

We have established an experimental mouse model of HC by the administration of DAB [13,14]. In this model, DAB metabolization generates an oxidative process, leading to inflammation and consequent liver injury reflected by foci of necrosis with decreased HO-1 immunohistochemical expression [14]. Under this experimental protocol, it was possible to identify early preneoplastic lesions which were characterized by proliferation of phenotipically altered hepatic foci (AHF) and oval cells after chronic exposure to DAB and to develop adenomas and hepatocarcinomas with an efficiency of 100% [14].

The aim of this work was to explore the liver capacity to restore homeostasis under carcinogen withdrawal analyzing the expression and cellular localization patterns of the cellular key adaptive protein HO-1 and to compare it with the continuous carcinogen administration. We have also investigated the expression of cell cycle related proteins and Bcl-2 during chemical induced hepatocarcinogenesis to evaluate whether the expression of these proteins was affected in different ways by deprivation of the carcinogen in this *in vivo *model.

## Methods

### Chemicals

Chemicals were reagent grade and purchased from Sigma Chemical Co. (St. Louis, MO, USA).

### Animals and treatment

**Figure 1:**
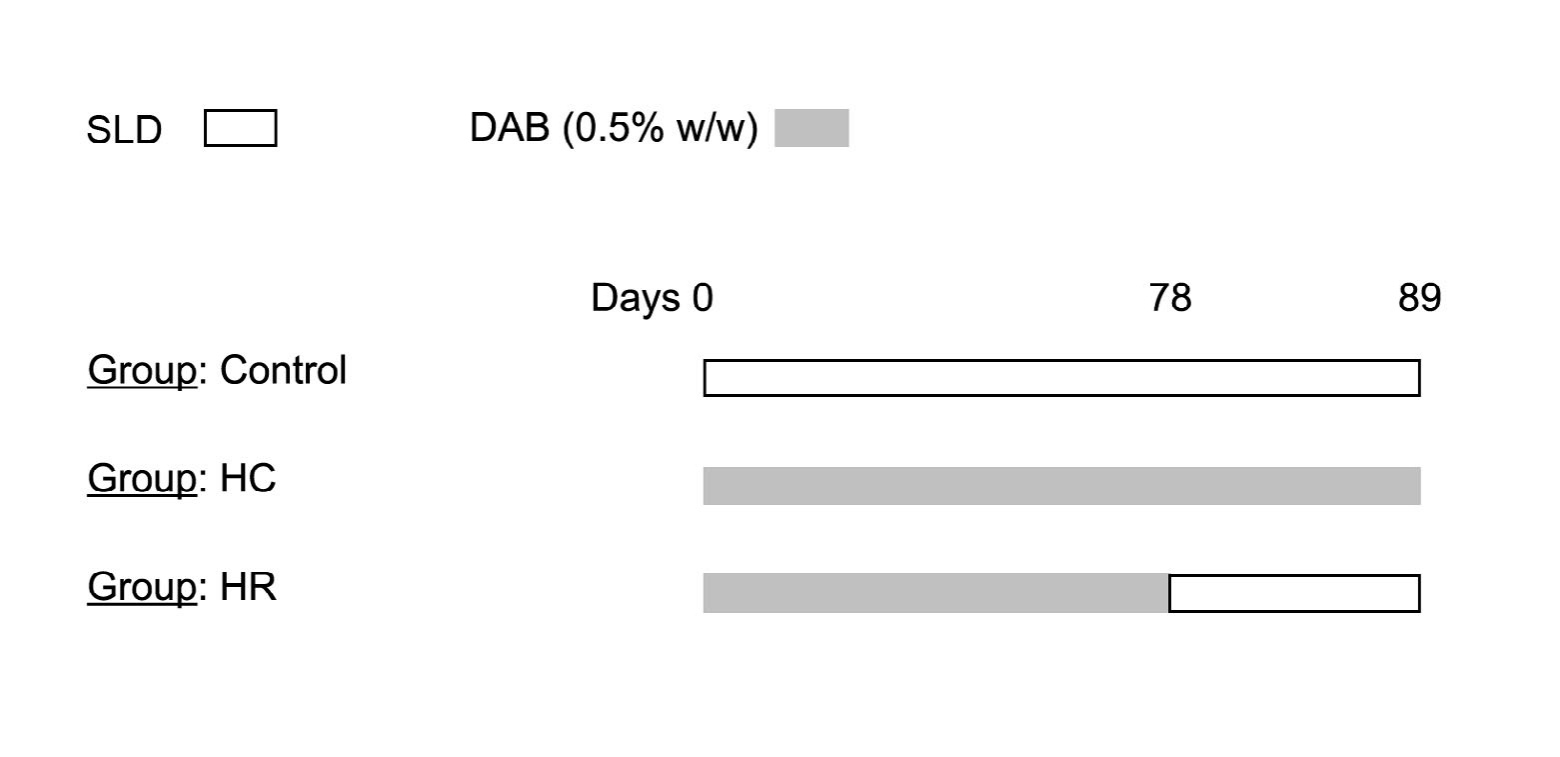
Experimental protocol.

Male CF1 mice (30 g) (n = 18) received a standard laboratory diet (SLD) (Purina 3, Asociación de Cooperativas Argentinas, San Nicolás, Buenos Aires, Argentina) supplemented or not with p-dimethylaminoazobenzene (DAB, 0.5 % w/w) under the intoxication protocol shown in Fig. 1. Animals with chemical induced hepatocellular carcinoma (HC group, n = 6) received the carcinogen during 89 days, when preneoplastic lesions were evident as previously reported [14]. The group of homeostatic response (HR, n = 6) received SLD since day 78; this time point was selected according to preliminary results demonstrating that optical proliferative morphological features were evident, indicative of a liver adaptive response by histological confirmation. Control animals (n = 6) received SLD for the whole period studied. All animals received food and water *ad libitum*.

Throughout the study, all animals were inspected at least twice daily. Body weight and food consumption were measured at intervals throughout the study. Food was removed from animals 16 hours before they were sacrificed under ether anesthesia at the indicated times.

All animals received human care and were treated in accordance with guidelines by the Animal Care and Use Committee of the Argentine Association of Specialists in Laboratory Animals (AADEALC) and in accordance with the UK Guidelines for the Welfare of Animals in Experimental Neoplasm (UK Coordinating Committee on Cancer Research, London, 1998).

### Protein extraction and western blotting

The protein extraction from liver tissue and western blot were performed as previously described in [10]. The following polyclonal antibodies were used: goat CDK2 (1:300), goat CDK4 (1:300), rabbit cyclin E (1:100), goat p21^cip1/waf1 ^(1:400), goat Bcl-2 (1:300) (Santa Cruz Biotech, Santa Cruz, CA, USA) and rabbit HO-1 (1:300) (Stressgen, Victoria, Canada). Goat actin (1:400) was used as a loading control. For quantification of immunoblots, relative intensity of bands was quantified by densitometry using Image Master Image Analysis software (Amersham-Pharmacia Biotech). Control for loading and transfer was obtained by probing with anti-β actin. The values of the different protein bands were normalized to the intensity of the loading control.

### Immunohistochemistry study

Complete necropsy of all animals was performed and liver samples were immediately processed. A random section, about 4 mm thick, was removed from the median, left lateral and right lateral lobes of the liver. This operation was repeated to examine samples from each liver. Additional sections were taken of grossly visible lesions not included within the above sections.

All sections were cut 3–5 μm thick from buffered formalin-fixed, paraffin-embedded tissue. After deparaffinization, sections were stained with haematoxylin-eosin (H&E), periodic acid-Schiff (PAS) reagent, Gomori's silver impregnation for reticulin and Prussian blue iron methods.

Liver foci as well as tumor histology were identified according to optical morphological published criteria [15].

Immunohistochemisty studies were performed in serial sections obtained from the paraffin blocks used for the histological diagnosis. They were deparaffinized with three 10 min. changes of xylene and then were hydrated in decreasing concentrations of ethanol and rinsed with water. Prior to staining, the slides were placed in 10 mM sodium citrate buffer pH 6 in a microwave oven (5 × 2 min at 750 W, with a cooling period of 6 min after each treatment).

Sections were pretreated with horse normal serum, diluted at 1:20, for 20 min. and then were incubated with: 1) a rabbit polyclonal antibody against HO-1, diluted at 1:500, overnight; 2) biotinylated anti-rabbit immunoglobulins serum diluted at 1:15 for 30 min and 3) alkaline phosphatase labeled streptavidin diluted at 1:15 for 30 min. Alkaline phosphatase activity was developed with the Fast-Red system (DAKO). Slides were lightly counterstained with H&E. All incubations were done at room temperature, and all washings were performed with phosphate-buffered saline buffer (pH 7.5)

Control sections used for determination of antibody reaction specificity: (a) tissue positive control (sections of mouse spleen) and (b) negative technique control (serial sections of each sample omitting the primary antibody).

The HO-1 immunostaining was qualitatively evaluated by the presence of positive or negative staining, in addition to the type of cells staining positive.

### Statistics

Statistical analysis was performed by Student's t-test. Results are presented as means ± SD and significance was defined as *P *< 0.05.

## Results

### Cyclin E and CDK2 protein levels are increased after carcinogen withdrawal

**Figure 2:**
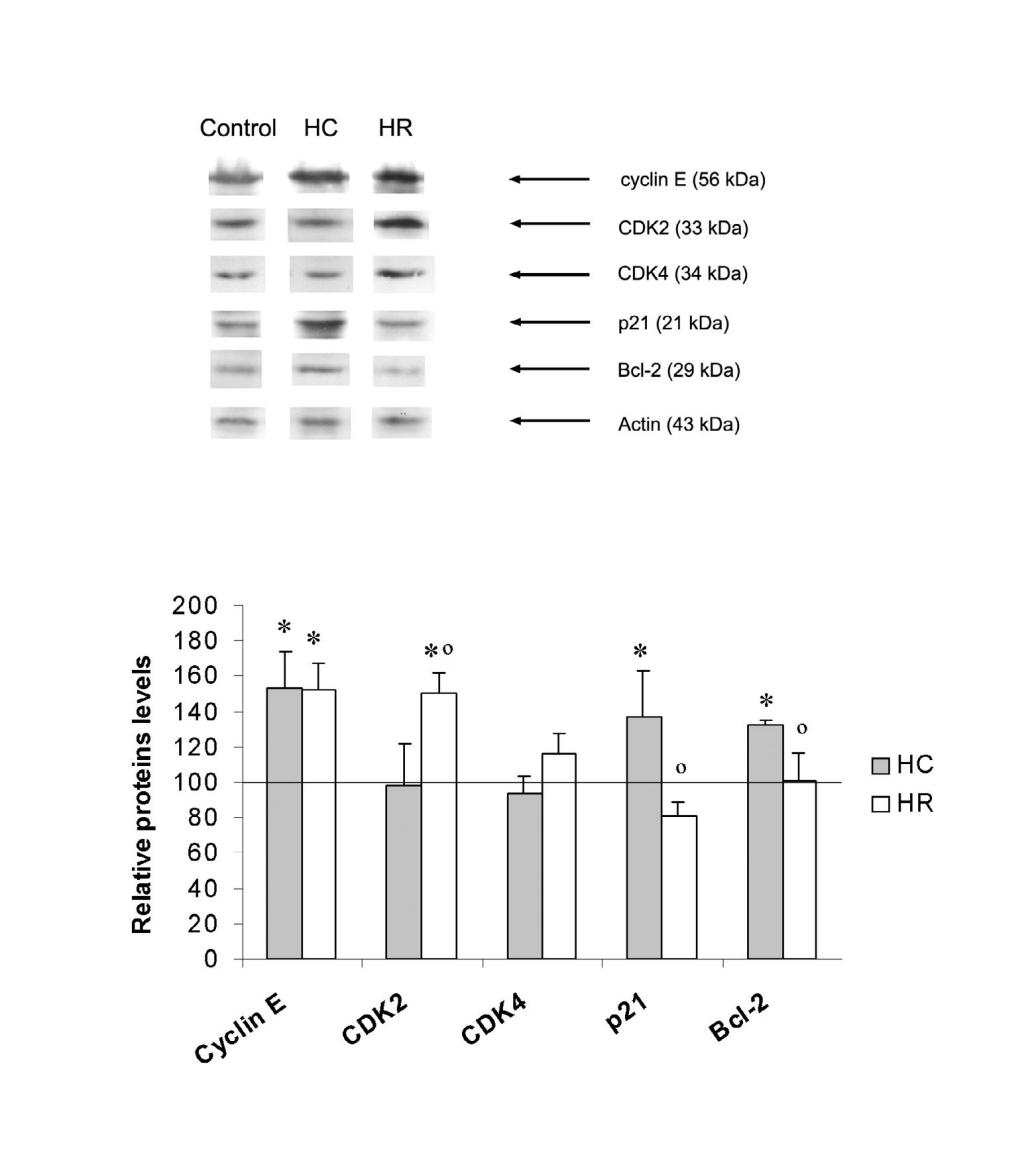
Expression of cyclin E, CDK2, CDK4, p21^cip1/waf1 ^and Bcl-2 in HR and during HC. Proteins were extracted from liver of control, HC and HR animals (six independent replicates). Equal quantities of proteins were loaded per lane. The proteins cyclin E, CDK2, CDK4, p21^cip1/waf1 ^and Bcl-2 were detected using polyclonal antibodies and visualized using ECL reagents. Intensity of bands was analyzed with Image Master. Protein expression levels were expressed as a percentage of control values, obtained from Western Blot analysis. *P < 0.05 *vs*. control group and °P < 0.05 *vs*. HC group.

Cell cycle progression is driven by activation of several CDKs that are regulated by CDK inhibitors. Hence, we analyzed the hepatic expression of cyclin E, CDK2, CDK4 and the cell cycle inhibitor p21^cip1/waf1 ^in animals receiving continuous dietary DAB (HC) and in the HR animals. HR was produced by carcinogen deprivation as described in Experimental Procedures. CDK2 expression increased in HR (49%, *P *< 0.05) respect to control and HC groups, while no variation was detected in CDK4 in any of the tested groups (Fig. 2). A prominent enhancement in cyclin E expression was observed in both HC (53%, *P *< 0.05) and HR (51%, *P *< 0.05), indicating that the induction of cyclin E expression provoked by the carcinogen could not be repressed by the carcinogen deprivation, at least during the assayed period. All together these results implicated cyclin E and its associated CDK (CDK2) in the regenerative process.

p21^cip1/waf1^, initially identified as a CDK inhibitor, plays an important role in the DNA damage response, participating in apoptosis, senescence, differentiation and cell cycle arrest. We have recently reported over expression of p21^cip1/waf1 ^in our HC model [10]. In the present work, we corroborated p21^cip1/waf1 ^induction (36%, *P *< 0.05) in animals receiving DAB continuously while carcinogen deprivation completely restituted p21^cip1/waf1 ^to basal levels (Fig. 2).

We evaluated the expression of the anti-apoptotic protein Bcl-2 in order to assess the involvement of the cell death machinery in the HR process. Bcl-2 expression slightly induced by DAB treatment (33%, *P *< 0.05) was completely reversed to control levels after DAB deprivation (Fig. 2); this finding correlates with the absence of apoptotic morphological features in immunohistochemical HR images.

Concluding, the deregulation in cell cycle progression seen in the tumor process as previously reported in our *in vivo *model of HC [10] was validated in the present work. Furthermore, we showed the abnormal expression of cyclin E and CDK2 in HR group which could be associated to the inflammatory process [16].

### HO-1 expression levels are restored after carcinogen withdrawal

**Figure 3:**
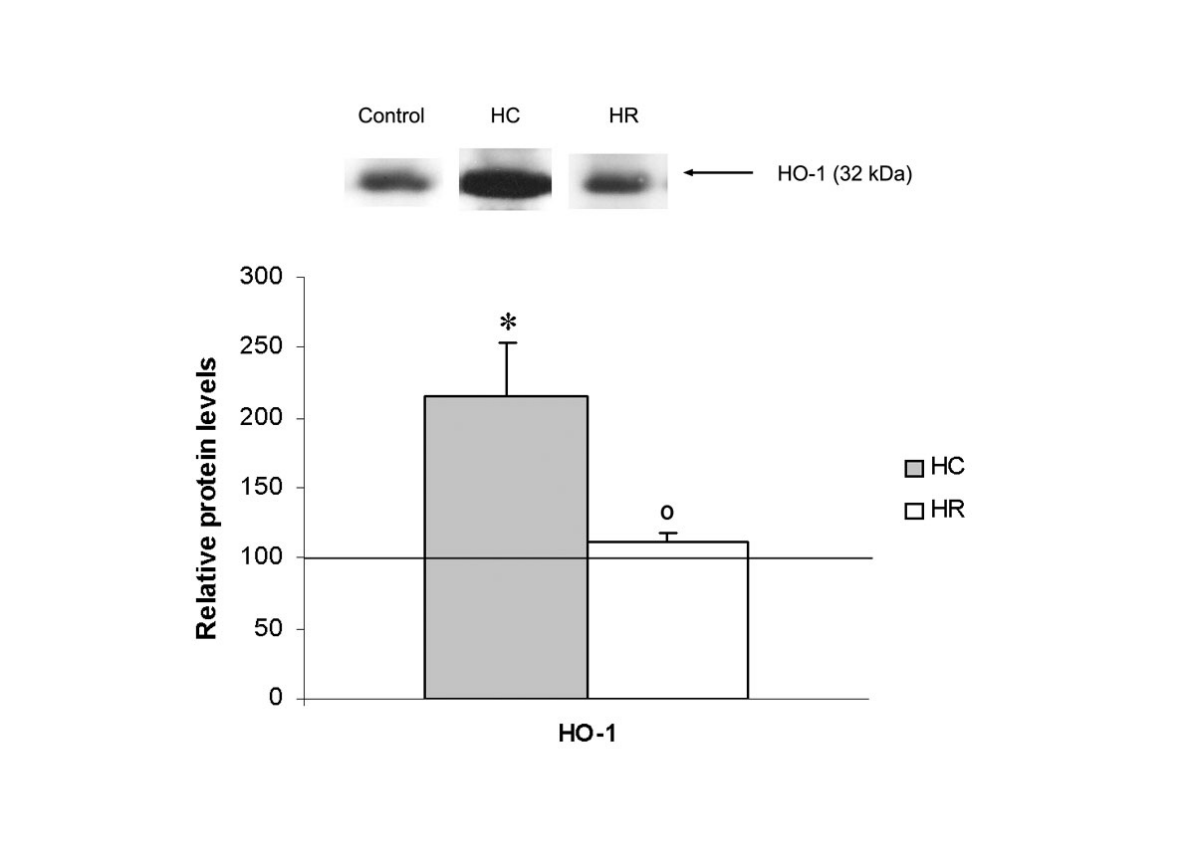
Expression of HO-1. Proteins were extracted from liver of controls, HC and HR animals (six independent replicates). Equal quantities of proteins were loaded per lane. HO-1 protein was detected using a polyclonal antibody and visualized using ECL reagents. Intensity of bands was quantified with Image Master. Protein expression levels were expressed as a percentage of control values. *P < 0.05 *vs*. control group and °P < 0.05 *vs*. HC group.

HO-1 is a stress inducible protein, useful to maintain cellular homeostasis [17,18]. We have previously reported increased HO-1 expression in DAB fed animals [10] as consequence of the oxidative stress provoked by the carcinogen [19]. Furthermore, we have studied this protein expression and cellular localization during the different stages of HC [14]. In the present work, we analyzed HO-1 expression in HR. Dietary carcinogen deprivation restored HO-1 expression levels to control values (Fig. 3) probably due to a decrease in the oxidative stress as result of carcinogen deprivation.

### HO-1 as an indicative marker of hepatic homeostatic response

**Figure 4:**
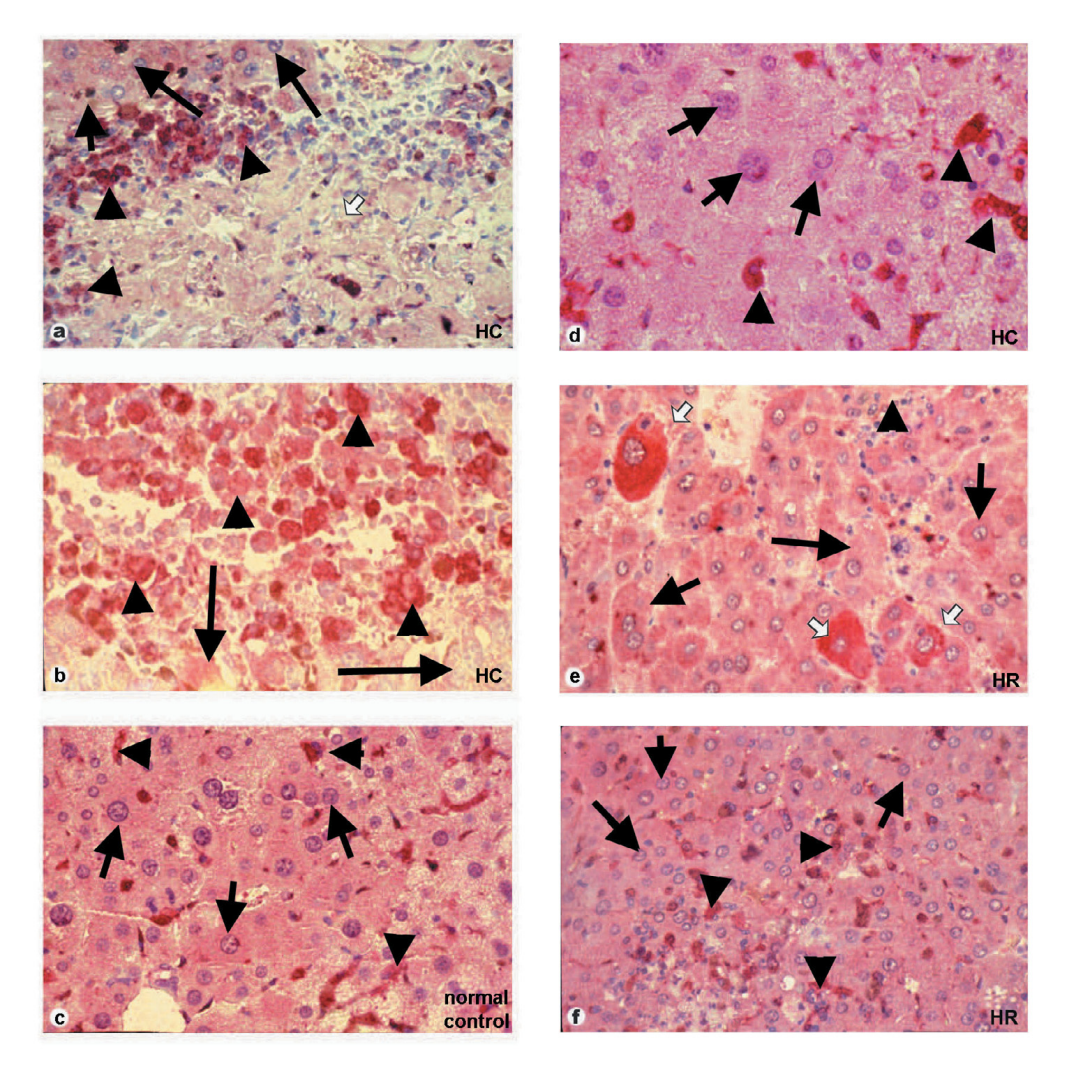
Representative findings of heme oxygenase (HO-1) immunoreactivity. Immunohistochemical results: **HC group**: a) non-apoptotic necrotic tissue foci (white broad arrow), regenerative and normal HO-1 positive hepatocytes (upper-left angle, black arrows) and b) hepatic macrophages hyperplasia (black arrows heads) and altered hepatocytes (black arrows); d) "altered hepatic foci lesions (AHF)" with decreased HO-1 expression central hepatocytes (black arrows) and surrounded by macrophages (black arrows heads). **HR group**: e) regenerative tissue foci with few intense staining rounded cells (white broad arrows), hepatocytes (black arrows) and few macrophages (black arrows heads) with similar normal tissue HO-1 expression; f) normal HO-1 expression and absence of tissue necrosis features in HR tissue hepatocytes (black arrows) and macrophages (black arrows heads). **Control group**: c) normal HO-1 hepatic tissue (black arrows) and macrophages (black arrows heads) expression. (Final Magnification × 312.5).

Considering that increase HO-1 expression in chemical HC plays an effective role to counteract oxidative damage and to control inflammation and confers marked resistance to apoptosis [10], we studied HO-1 expression by immnunohistochemical analysis in the liver of animals under a protocol of carcinogen withdrawal. The HC group showed: necrotic tissue foci with mild (-/+) or absence (-) of HO-1 immunoreaction surrounded by regenerative and normal HO-1 positive (++/+++) hepatocytes (Fig. 4a upper-left angle) and hepatic macrophages hyperplasia with increased HO-1 immunoreaction (+++) (Fig. 4b) ; morphological and immunohistochemical "altered hepatic foci lesions" (AHF) with central disposed large irregular hepatocytes with decreased (-/+) HO-1 expression and intense (+++) immunostaining periphery hepatic macrophages (Fig. 4d). The HR group showed: regeneration tissue foci with few intense (+++) immunostaining hypertrophic rounded cells (Fig. 4e) and the remaining hepatocytes with HO-1 expression similar (+/++) to the normal control group (Fig. 4c), and absence of tissue necrosis features with normal (+/++) HO-1 expression (Fig. 4f).

It is evident that HO-1 inmmunoreactivity showed a variable pattern in the regenerative tissue with altered expression associated with cell morphological and size changes. We propose that this protein over expression in the normal liver could be indicative of HO-1 protective function against the inflammatory injury provoked by the carcinogen.

## Discussion

Disruption of the regulatory system controlling G_1 _phase progression is a common event in human hepatocarcinogenesis [9]. Oxidative injury may produce cell death and a compensatory increased in cell proliferation. We have previously demonstrated that DAB treatment leads to deregulation of the cell cycle progression [10].

Members of the Bcl-2 family of proteins are important regulators of the programmed cell death pathway [20] with individual members that can suppress (eg Bcl-2, Bcl-XL) or promote (eg Bax, Bad) apoptosis. While the mechanism(s) of Bcl-2's anti-apoptotic function is not yet clear, introduction of Bcl-2 into most eukaryotic cell types protect the recipient cell from a wide variety of stress applications that lead to cell death [21]. Although, over expression of the anti-apoptotic protein Bcl-2 in HC previously reported [10] was confirmed, basal levels of this protein were detected in HR, suggesting that apoptosis could be triggered under regenerative conditions to restore homeostasis.

We have demonstrated previously that DAB treatment leads to deregulation of the cell cycle with remarkable over expression of cyclin E [10]. Cyclin E over expression is a common feature in cancer [22], suggesting that cyclin E/CDK2 deregulation contributes to tumorigenesis. However, uncontrolled cell proliferation and abnormal expression of cell cycle related proteins are observed in inflammatory processes [16] suggesting that cell proliferation is required in the regenerative process. We reported here a striking increase in CDK2 and cyclin E expression in HR animals.

HO-1 is an inducible and ubiquitous 32 kDa isoform highly expressed in spleen and liver and normally found in very low levels in mammalian tissues [23]. The regulation of its potent enzymatic activity depends primarily on the control of HO-1 expression at transcriptional level [23,25]. HO-1 induction is considered to be an adaptative cellular response to oxidative stress [26,19]. Up-regulation of HO-1 expression in DAB fed animals, as an endogenous antioxidant defense mechanism, would serve as a protective factor controlling the changes associated to the carcinogenic process [19,14]. We demonstrated that HO-1 levels were diminished in HR group in comparison with animals that received a continuous carcinogenic diet, indicating that deprivation of the carcinogen will promote hepatic regeneration and enable to restore the hepatic homeostasis. Increase HO-1 expression in chemical HC plays an effective role to counteract oxidative damage and to control inflammation and confers marked resistance to apoptosis [10]. HO-1 induction or HO-1 gene transfer reduces apoptosis with an increase in Bcl-2 in rat ischemic hearts [27] and liver grafts [28]. In agreement with these data, we observed in HC the same profile of correlation between Bcl-2 and HO-1. The mechanism by which HO-1 induces over expression of Bcl-2 is yet unclear. The present paper shows a decrease in both HO-1 and Bcl-2 expression in HR model, restoring the apoptotic machinery functionality.

One important component observed by immunohistochemical studies in the liver tissue slides of HR animals, was the presence of sinusoidal lining fixed macrophages (Kuppfer's cells) as well as free macrophages surrounding foci of necrosis and nodular lesions, as indicative of an inflammatory response. Also, regeneration cells displayed changes in type, size and intensity of HO-1 immunostaining. Over expression of this protein, stimulates the proliferation and cell differentiation and promotes angiogenesis [29,30], related to an inflammatory reaction [31,32].

Finally, for limited resections (less than 60–70%) or moderate hepatocytes damage, the rate of regeneration is sufficient enough to restore the greater part of the liver mass and function. In this context, we might consider HO-1 over expression as an alternative therapy to diminish the tissue inflammation and also its deterioration in many illnesses.

HO-1 has also been identified as a key enzyme for the cytoprotection of many cell types. Increased expression of HO-1 has been shown to be protective in ischemia/reperfusion injury, in organ transplantation, in protection against renal and pulmonary injury, and in amelioration of adverse hemodynamic effects resulting from liver disease and portal hypertension [33-35]. Even more, it was recently reported that hepatitis C (HCV) infection increased HO-1 mRNA expression and protein levels in human liver cells and was suggested to likely represent a protective response against possible oxidative or other insult from HCV proteins [36]. Thus, Ghaziani et al [36] suggested that HO-1 importance extends beyond its function in the catabolism of heme.

## Conclusion

Taken together, these results show that the regenerative capacity of the liver is still observed in the pre-neoplastic tissue after carcinogen withdrawal suggesting that reversible mechanism/s arise to compensate necrosis and restitute homeostasis. Furthermore, these results provide a rationale for using HO-1 over expression as an alternative therapy to diminish tissue inflammation and tissue deterioration in many illnesses.

## Competing interests

The author(s) declare that they have no competing interests.

## Authors' contributions

CC and PS carried out the protein extraction and western blot analysis, they also performed the statistical analysis and drafted the manuscript. RM carried out the immunohistochemistry and drafted the manuscript. FC administered the carcinogen to the animals. AB drafted the manuscript. EV conceived, designed & coordinated the study and corrected the manuscript.

All the authors read and approved the final manuscript.

## Pre-publication history

The pre-publication history for this paper can be accessed here:
